# Determination of Activation Energies and the Optimum Temperatures of Hydrolysis of Starch by *α*-Amylase from Porcine Pancreas

**DOI:** 10.3390/molecules26144117

**Published:** 2021-07-06

**Authors:** Justyna Miłek

**Affiliations:** Department of Chemical and Biochemical Engineering, Faculty of Chemical Technology and Engineering, University of Science and Technology in Bydgoszcz, Seminaryjna 3, 85-326 Bydgoszcz, Poland; jmilek@utp.edu.pl; Tel.: +48-52374-90-49

**Keywords:** porcine pancreas *α*-amylase, activation energy, deactivation energy, optimum temperature

## Abstract

The present paper reports the determination of the activation energies and the optimum temperatures of starch hydrolysis by porcine pancreas *α*-amylase. The parameters were estimated based on the literature data on the activity curves versus temperature for starch hydrolysis by α-amylase from porcine pancreas. It was assumed that both the hydrolysis reaction process and the deactivation process of *α*-amylase were first-order reactions by the enzyme concentration. A mathematical model describing the effect of temperature on porcine pancreas *α*-amylase activity was used. The determine deactivation energies *E_a_* were from 19.82 ± 7.22 kJ/mol to 128.80 ± 9.27 kJ/mol, the obtained optimum temperatures *T_opt_* were in the range from 311.06 ± 1.10 K to 326.52 ± 1.75 K. In turn, the values of deactivation energies *E_d_* has been noted in the range from 123.57 ± 14.17 kJ/mol to 209.37 ± 5.17 kJ/mol. The present study is related to the starch hydrolysis by *α*-amylase. In the industry, the obtained results the values *E_a_*, *E_d_*, *T_opt_* can be used to design and optimize starch hydrolysis by *α*-amylase porcine pancreas. The obtained results might also find application in research on the pharmaceutical preparations used to treat pancreatic insufficiency or prognosis of pancreatic cancer.

## 1. Introduction

The starch molecules are glucose polymers linked together by *α*-1,4 and *α*-1,6 glucosidic bonds. Starch is insoluble in water at room temperature [1,2]. These products are the source of complex carbohydrates. They are synthesized naturally in a variety of plants. Plants with a high starch content include corn, potato, rice, sorghum, wheat, cassava and rhizome and bulbil of Chinese jam. Starchy substances are a major part of the human diet for most people in the world, as well as many other animals [1,3].

The enzymes α-amylases (E.C. 3.2.1.1) catalyze the hydrolysis of *α*-1,4 glycosidic bonds present in starch, glycogen and other related carbohydrates to low molecular weight products, such as glucose, maltose and maltotriose [4,5,6,7,8]. These enzymes are present in plants, animals and microorganisms [9] and have extensive applications in medicine [10,11,12,13,14], textiles [11], detergent [11], fermentation [11] and the food industry [4,11].

Amylases have potential application in various branches of industrial processes [7] and have been used in baking [9,11], brewing [9,11] and saccharification of starch.

During the baking process, gelatinization of the starch granules occurs, which together with the hydrolysis of the starch by *α*-amylase to cause its liquefaction [9]. In beer, brewing is the process-mashing (malting) in which enzymatic degradation of starch into fermentable sugars (maltose) occurs by inter alia α-amylase [9].

The saccharification of starch is an enzymatic hydrolysis of starch byα-amylase which takes place in three stages. The first is gelling, which is aimed at dissolving the starch granules. In turn, the second step consists of partial hydrolysis of the suspension, thus, it may lead to reduce its viscosity. The final stage of depolymerization is mainly the formation of mono-, di- and tri-saccharides. This process is called saccharification, due to the formation of saccharides [3].

The *α*-amylase which is extremely similar to human pancreatic *α*-amylase and is often used in industry is the three-dimensional structure of porcine pancreatic (*Sus scrofa*). Molecular cloning and primary structure analysis of porcine pancreatic *α*-amylase showed the highest homology to the human pancreatic *α*-amylase sequence (87.1%) among all the amylases known [15].

In addition to industrial use, *α*-amylase from porcine pancreas is used for example in health food research [16,17,18], to assay resistant starch (RS), not broken down by human enzymes in the small intestine [19] and is also used in medical diagnostics [12,13,14]. One of the problems is effective diagnostic methods allowing for the prognosis of pancreatic cancer of cancer, which is an exceptionally aggressive tumour with high mortality. Stotz et al. [12] hypothesized that the level of *α*-amylase and lipase quantities in the peripheral blood and the calculation of the lipase and *α*-amylase ratio at the time of localized pancreatic cancer might represent a novel marker for individualized patient risk assessment pancreatic cancer.

The study of *α*-amylase activity, important in the diagnosis of pancreatic cancer in humans, is also used in the industrial hydrolysis of starch by *α*-amylase. The processes involving *α*-amylase cannot be designed without knowing the kinetic parameters of the process. Therefore, studies on the effect of temperature on *α*-amylase activity are required. Hydrolysis with porcine pancreas *α*-amylase is usually carried out at temperatures higher than 310 K [7,16,20,21,22,23,24,25], thus, a significant inactivation of the enzyme may occur. Therefore, it is necessary to determine the activation energy *E_a_*, the deactivation energy *E_d_* and the optimum temperature *T_opt_* for porcine pancreas *α*-amylase. The determination of parameters *E_a_*, *E_d_*, *T_opt_* based on experimental data on the effect of temperature on the activity of *α*-amylase from porcine pancreas has not been presented in previous studies.

The purpose of the present work was to estimate parameters of the activation energies *E_a_*, the deactivation energies *E_d_* and the optimum temperatures of starch hydrolysis by pancreas *α*-amylase, whose obtained values can be used in works focused on prognosis for a pancreatic tumour or in industrial purposes can be used to designed and optimized starch hydrolysis by *α*-amylase porcine pancreas.

## 2. Results and Discussion

**Figure 1:**
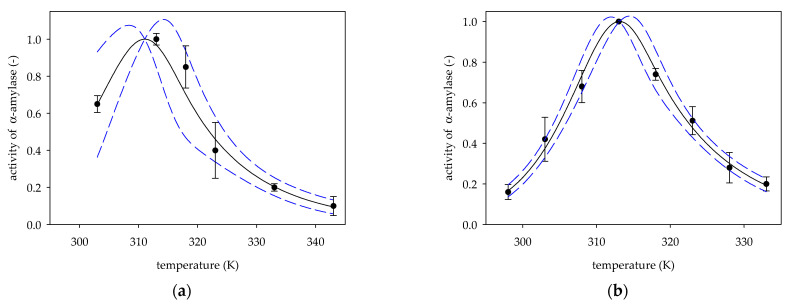
The activity of *α*-amylase porcine pancreas by measurements: (**a**) Akhond et al. [7]; (**b**) Aksoy et al. [20]; (●) experimental data, (solid line) Equation (6); (dotted line) 95% confidence band.

**Figure 2:**
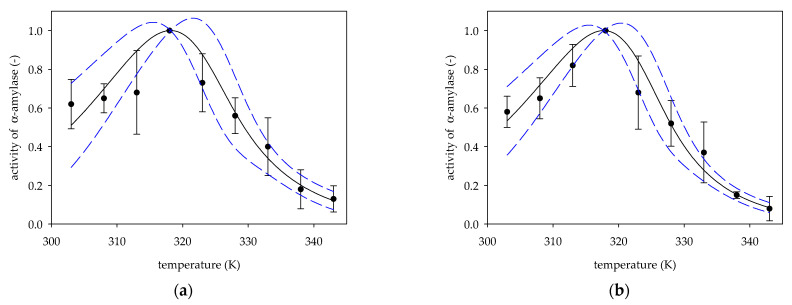
The activity of *α*-amylase porcine pancreas by measurements Gopal et al. [15]: (**a**) isoform I; (**b**) isoform II; (●) experimental data, (solid line) Equation (6); (dotted line) 95% confidence band.

**Figure 3:**
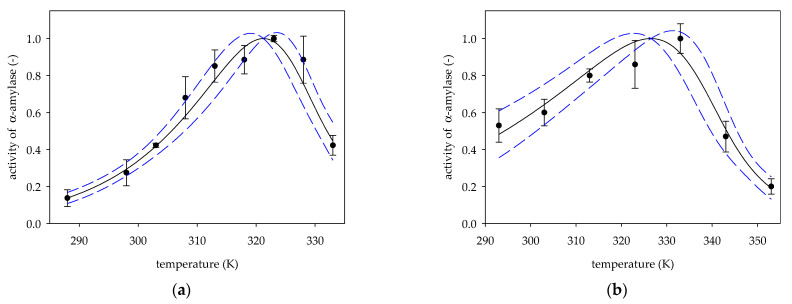
The activity of *α*-amylase porcine pancreas by measurements: (**a**) Louati et al. [21]; (**b**) Guoet al. [22]; (●) experimental data, (solid line) Equation (6); (dotted line) 95% confidence band.

Based on experimental data on the change in the activity of α-amylase from the porcine pancreas [7,16,20,21,22] vs. temperature, values of deactivation energies *E_d_*, *β* parameters and temperatures optimal *T_opt_* were determined from Equation (6). Figure 1, Figure 2 and Figure 3 show experimental data on α-amylase activity by hydrolysis of starch as a substrate, along with activity curves plotted based on Equation (6) for the values of the specified parameters *E_d_*, *T_opt_*, *β* listed in Table 1.

The obtained parameters *T_opt_*, *β*, *E_d_* for *α*-amylase from porcine pancreas are presented in Table 1, according to the increasing value of the temperatures optimal *T_opt_*. Then, based on the value of the deactivation energy *E_d_* and the parameter *β*, the activation energy value *E_a_* was calculated based on Equation (8). The obtained *E_a_* values are presented in Table 1.

In addition, Figure 1, Figure 2 and Figure 3 present standard deviation errors for experimental points, while the 95% confidence limits were marked for the obtained curves.

Table 2 presents statistical data obtained during the determination of the parameters of porcine pancreatic *α*-amylase. High values of regression coefficient *R*^2^ (above 0.93) and standard errors of estimation *RSS* below 0.19 were obtained; while statistical variability of *E_d_* and *T_opt_* parameters in most of the analyzed cases *p* < 0.0001. *F*-Fisher test values were from 44.93 to 170.77 with a low probability value [*p* ≤ 0.0031] which confirmed, that when determining the parameters, it was appropriated to apply Equation (6).

This work aimed to identify the activation energy *E_a_*, the deactivation energy *E_d_* and the optimum temperature *T_opt_* of starch hydrolysis by porcine pancreas *α*-amylase. Knowing the obtained values can be used in works focused on prognosis for a pancreatic tumour.

### 2.1. The Activation Energy E_a_

The obtained values of the activation energy *E_a_* of starch hydrolysis by *α*-amylase from porcine pancreas were in the range from 19.82 ± 7.22 kJ/mol to 128.80 ± 9.27 kJ/mol. 

In turn, in [23,24] determined values of *E_a_* activation energy of the starch hydrolysis were equal to 48.91 kJ/mol and 50.16 kJ/mol, respectively.

The analysis of the data presented in Table 2 allows concluding that there is a correlation between the values of the activation energy *E_a_*, deactivation energy *E_d_* and parameter *β*. Indeed, it has been reported that for most of the analyzed cases, with the simultaneous increase in the values of parameter *β* and *E_d_* increase, the values of *E_a_* increase. The longer the starch hydrolysis by α-amylase is carried out, the lower the value of the *β* parameter. Determining the influence of the time of measurement α-amylase activity on the value *β* parameter of will be the aim of further research.

According to the calculations for the measurement performed by Aksoy et al. [20], the energy value *E_a_* was six as high compared to the *E_a_* values obtained by Guo et al. [22]. The observed difference may be due to the different time at which the α-amylase activity is determined. Indeed, the measurements time of hydrolysis starch time was equal to 5 min and 30 min in the studies of Aksoy et al. [20] and Guo et al. [22], respectively.

Additionally, α-amylase from porcine pancreas used in to study by Guo et al. [22] was from Shanghai Kaiyang Biological Technology Co (Shanghai, China). The highest *E_a_* value was obtained for *α*-amylase from Merck AG (Darmstadt, Germany) for measurements carried out by Aksoy et al. [20].

### 2.2. The Activation Energy of the Deactivation Process E_d_

In the present study, the obtained values of the deactivation energy process were, in the range from 123.57 ± 14.17 kJ/mol to 209.37 ± 5.17 kJ/mol (Table 2). 

The lowest value *E_d_* was obtained in the hydrolysis starch by *α*-amylase from porcine pancreas from Biological Technology Co., Ltd. (Shanghai, China) for measurements Guo et al. [22]. Simultaneously, the *E_a_* value for this α-amylase was also the lowest. The lowest *E_d_* value was obtained for *α*-amylase from Merck AG (Germany) for measurements Aksoy et al. [20]. In addition, the *E_a_* value for this *α*-amylase was the highest.

The differences in the obtained activation energy value of the deactivation process *E_d_* can be caused by the use of α-amylase from a different company.

### 2.3. Optimum Temperature T_opt_

The determined values of the optimum temperature *T_opt_* of starch hydrolysis by *α*-amylase from porcine pancreas were different by about fifteen degrees and are in the range from 311.06 ± 1.10 K to 326.52 ± 1.75 K (Table 2). The highest *T_opt_* value was obtained in the hydrolysis starch by *α*-amylase from porcine pancreas from Biological Technology Co., Ltd. (Shanghai, China) for measurements Guo et al. [22]. The lowest value *T_opt_* was obtained in the hydrolysis starch by *α*-amylase from porcine pancreas from Sigma-Aldrich for measurements Akhond et al. [7]. Additionally, it should be noted that the measurement was performed in the shortest time, i.e., 3 min.

In works [23,24], a *T_opt_* of starch hydrolysis by porcine pancreatic *α*-amylase were presented and equals 313 K and 327 K, respectively.

## 3. Materials and Methods

### 3.1. The Effect of Temperature on α-Amylase Activity

The value of activation energy, *E_a_* can be determined from the curve of the dependence of the logarithm of the reaction rate (ln *v*) on the reciprocal of temperature (1/*T*), the so-called Arrhenius dependence [23,26]. However, the determined values of *E_a_* and *E_d_* by application of the traditional method is burdened with an error. Many researchers have studied the kinetic parameters of α-amylase of other origins [24,26]; however, the parameters *T_opt_*, *E_a_* and *E_d_* were not obtained simultaneously for *α*-amylase porcine pancreas.

When studying *α*-amylase activity during the hydrolysis of starch, it is assumed that the change in substrate concentration *S* during reaction time *t* describes by the first-order equations due to the concentration of the enzyme

(1) $$\frac{dS}{dt}=-kE,$$

where *k* is the kinetic constant of the enzymatic reaction (1/min) and *E* is the concentration of the active enzyme (M). 

The change in *α*-amylase dimensionless activity *a* is also described by the first-order kinetics [26,27] with the following equation

(2) $$\frac{da}{dt}-k_{d}a,$$

where *k_d_* is the kinetic constant of the enzymatic reaction (1/min).

The solution of Equation (2) for the initial condition *a* (*t =* 0) *=* 1 is

(3) $$a=\exp\left(-k_{d}t\right)=f\left(t\right),$$

Kinetic constants *k* and deactivation constant *k_d_* depend on temperature *T* and are described by the Arrhenius equations as:

(4) $$k\left(T\right)=A\exp\left(-\frac{E_{a}}{RT}\right),$$

(5) $$k_{d}\left(T\right)=B\exp\left(-\frac{E_{d}}{RT}\right),$$

where A,B  are pre-exponential factors of the hydrolysis reaction rate or deactivation process of *α*-amylase (1/min), *E_a_* is the activation energy for the enzymatic reaction (kJ/mol) while *E_d_* is the activation energy of the deactivation process (kJ/mol), *R* is the gas constant 8.315 (J/(mol·K)) and *T* is the absolute temperature (K).

Equations (1)–(5) are the basis for the derived dependence of the change in the dimensionless activity of the enzyme on the temperature measurement *T* as follows:

(6) $$a\left(T\right)=\frac{\exp\left(\frac{\left(T_{opt}-T\right)}{RTT_{opt}}\cdot \frac{E_{d}\beta }{\left(\exp\beta -1\right)}\right)\left\{1-\exp\left[-\beta \exp\left(\frac{E_{d}\left(T-T_{opt}\right)}{RTT_{opt}}\right)\right]\right\}}{1-\exp\left(-\beta \right)},$$

where *T_opt_* is the temperature at which *α*-amylase shows maximum activity (K) and dimensionless parameter *β* determines the relationship

(7) $$\beta =Bt_{a}\exp\left(-\frac{E_{d}}{{RT}_{opt}}\right),$$

where *t_a_* is the reaction time of starch hydrolysis by *α*-amylase from porcine pancreas (min).

The full analysis of the solution of Equation (6) was presented in an earlier publication of Wojcik and Miłek [28].

It means that knowing the value of the activation energy of the deactivation reaction *E_d_* and the parameter *β*, the activation energy *E_a_* is determined by the following equation

(8) $$E_{a}=E_{d}-\frac{E_{d}\cdot \beta }{\exp\beta -1},$$

Equations (6)–(8) were used to determine the kinetic parameters of inulin hydrolysis by exo-inulinases *Aspergillus niger* [29], olive oil hydrolysis by porcine pancreas lipase [30,31], p-nitrophenyl palmitate hydrolysis by lipases from *Rhizopus oryzae* 3562 and *Enterobacter aerogenes* [32], hydrolysis of starch by α-amylase *Bacillus licheniformis* [33], inulin hydrolysis by endo-inulinase *A. niger* [34] and inulin hydrolysis by inulinase *K. marxianus* [28].

Based on Equation (6), the parameters $E_{d}$, *β* and *T_opt_* were estimated by non-linear regression according to the methods of least squares [25,27,35,36,37] determining the residual sum of squared (*RSS*) from the equation:

(9) $$RSS\left(E_{d},\beta ,T_{opt}\right)=\overset{n}{\underset{i=0}{\sum }}\frac{1}{a_{\exp}{}^{2}}{\left(a_{\exp}-a_{cal}\left(E_{d},\beta {,T}_{i}{,T}_{opt}\right)\right)}^{2}=min,$$

where $a_{\exp}$ is *α*-amylase dimensionless activity determined experimentally and $a_{cal}\left(E_{d},\beta {,T\;,T}_{opt}\right)$ is *α*-amylase dimensionless activity calculated from Equation (6).

### 3.2. Assay of α-Amylase Activity

Literature data [7,16,20,21,22] for porcine pancreas *α*-amylase from various companies have been analyzed.

Amylase activity was determined according to Miller [38]. The reaction mixture consisted of starch, buffer and enzyme solution and incubated for different time (min) at 90 °C. The reaction was stopped by the addition of 3,5 dinitrosalicylate (DNS) reagent. The quantity of reducing sugar was measured spectrophotometrically. The unit of amylase was defined as the amount of enzyme which produced 1 μmol of reducing sugar as glucose in 1 min underspecified condition.

Table 3 presents the conditions for measuring *α*-amylase activity during the hydrolysis of starch with the various buffer pH, the various duration of measurement and the used the initial concentration of starch [7,16,20,21,22].

## 4. Conclusions

The following method of determining parameters was used: the optimum temperatures *T_opt_*, activation energies *E_a_* and deactivation energies *E_d_* of olive oil hydrolysis by *α*-amylase from porcine pancreas reaction based on four curves of changes activity of *α*-amylase from porcine pancreas depending on the temperature of hydrolysis. For the optimum temperatures *T_opt_*, the difference between the obtained values is fifteen degrees. The differences in the calculated values of the deactivation energy *E*_d_ are equal to about 110 kJ/mol, for the activation energy of the reaction *E*_a_ equal to about 85 kJ/mol. The reason for the differences in the obtained values *E_a_*, *E_d_*, *T_opt_* is, above all, different origins of *α*-amylase from porcine pancreas. The lowest values *E_a_* and *E_d_*, together with the highest *T_opt_* was obtained for the enzyme derived from Shanghai Kaiyang Biological Technology Co., Ltd. The highest values *E_a_* and *E_d_*, together with lower *T_opt_* obtained for an enzyme derived from Merck AG (Germany). Additionally, it is essential to mention that the noted differences in values of parameters can be caused by the various duration of the *α*-amylase activity assay, different pH values of the hydrolyzed starch used to test the *α*-amylase activity as well as different concentrations of the starch.

The obtained results the values *E_a_*, *E_d_*, *T_opt_* can be used to design and optimize starch hydrolysis *α*-amylase by porcine pancreas in the food, pharmaceutical and industrial industries, among others.

## Figures and Tables

**Table 1:** The values of kinetic parameters estimated for *α*-amylase porcine pancreas.

Figure	*t* (min)	$T_{\mathrm{opt}}\;(K)$	$E_{d}\;(\mathrm{kJ}/\mathrm{mol})$	*β*	$E_{a}\;(\mathrm{kJ}/\mathrm{mol})$	$E_{d}/E_{a}\;$	Ref.
1a	3	311.06 ± 1.10	164.9 ± 19.14	1.46 ± 0.29	92.08 ± 23.07	1.79	[7]
1b	5	313.12 ± 0.55	209.37 ± 5.17	1.68 ± 0.12	128.80 ± 9.27	1.63	[20]
2a	60	318.17 ± 1.36	152.83 ± 11.06	0.83 ± 0.24	54.75 ± 17.02	2.79	[15]
2b	60	317.74 ± 1.04	164.06 ± 9.23	0.71 ± 0.16	51.41 ± 12.71	3.19	[15]
3a	15	321.24 ± 1.04	162.70 ± 19.21	0.76 ± 0.17	54.07 ± 15.88	3.01	[21]
3b	30	326.52 ± 1.75	123.57 ± 14.17	0.34 ± 0.10	19.82 ± 7.22	6.23	[22]

**Table 2:** The statistical data obtained by determining the kinetic parameters of *α*-amylase porcine pancreas.

Figure	*R* ^2^	*RSS*	*p*			*F*	*P*	Ref.
			$E_{d}\;(\mathrm{kJ}/\mathrm{mol})$	$T_{\mathrm{opt}}\;(K)$	*β*			
1a	0.9789	0.1370	0.0033	<0.0001	0.0151	69.56	0.0031	[7]
1b	0.9856	0.0080	<0.0001	<0.0001	<0.0001	170.77	<0.0001	[20]
2a	0.9374	0.1912	<0.0001	<0.0001	0.0132	44.93	0.0002	[15]
2b	0.9679	0.1592	<0.0001	<0.0001	0.0041	90.43	<0.0001	[15]
3a	0.9817	0.1034	0.0001	<0.0001	0.0044	160.97	<0.0001	[21]
3b	0.9751	0.1117	0.0010	<0.0001	0.0185	78.41	0.0006	[22]

**Table 3:** Conditions for measuring *α*-amylase porcine pancreas activity.

BufferpH	*t* (min)	λ (nm)	Concentration of Starch	Source Inulinase	Ref.
6.9 sodium phosphate	3	540	0.5%	Sigma-Aldrich (St. Louis, MO, USA)	[7]
6.9 phosphate	5	600	2%	Merck AG (Germany)	[20]
6.9 sodium phosphate	60	540	1%	Sigma Chemical Company	[16]
7.0 MOPS	15	575	0.5%	Sigma	[21]
7.0 MES	30	520	-	Shanghai Kaiyang BiologicalTechnology Co., Ltd. (Shanghai, China)	[22]

## Data Availability

All data are contained within the article.
